# Feather mites (Acariformes, Astigmata) from marine birds of the Barton Peninsula (King George Island, Antarctica), with descriptions of two new species

**DOI:** 10.3897/zookeys.1061.71212

**Published:** 2021-10-04

**Authors:** Yeong-Deok Han, Sergey V. Mironov, Jeong-Hoon Kim, Gi-Sik Min

**Affiliations:** 1 Department of Biological Sciences, Inha University, Incheon 22212, Republic of Korea Inha University Incheon Republic of Korea; 2 Restoration Assessment Team, Research Center for Endangered Species, National Institute of Ecology, Gowol-gil 23, Yeongyang-gun, 36531, Republic of Korea National Institute of Ecology Yeongyang-gun Republic of Korea; 3 Zoological Institute, Russian Academy of Sciences, Universitetskaya embankment 1, Saint-Petersburg, 199034, Russia Zoological Institute, Russian Academy of Sciences Saint-Petersburg Russia; 4 Korea Polar Research Institute, Yeonsu-gu, Incheon 21990, Republic of Korea Korea Polar Research Institute Incheon Republic of Korea

**Keywords:** *Alloptes*, Analgoidea, Antarctica, feather mites, *Ingrassia*, systematics

## Abstract

We report on the first investigation of feather mites associated with birds living on the Barton Peninsula (King George Island, Antarctica). We found seven feather mite species of the superfamily Analgoidea from four host species. Two new species are described from two charadriiform hosts: Alloptes (Sternalloptes) antarcticus**sp. nov.** (Alloptidae) from *Stercorariusmaccormicki* Saunders (Stercorariidae), and *Ingrassiachionis***sp. nov.** (Xolalgidae) from *Chionisalbus* (Gmelin) (Chionidae). Additionally, we provide partial sequences of the mitochondrial cytochrome c oxidase subunit I (COI), which was utilized as a DNA barcode, for all seven feather mite species.

## Introduction

Feather mites (Astigmata, Analgoidea and Pterolichoidea) are a vast group of highly specialized parasites or mutualistic ectosymbionts that spend their entire life cycle on their bird hosts (Gaud and Atyeo 1996; Dabert and Mironov 1999; Proctor 2003). Most of these mites occupy various microhabitats in the plumage of birds; however, representatives of a few families are parasites located on the skin and in the respiratory tract of their avian hosts. Owing to their specialization to particular microhabitats on birds and dispersal mainly by a direct contact of host individuals, feather mites usually show a high level of host-specificity (Mironov and Dabert 1999; Proctor and Owens 2000; Dabert 2005).

Antarctica is the fifth largest and most isolated continent on our planet (Peck 2018; Sancho et al. 2019). On this 14 million km^2^ continent, less than 0.35% of the territory remains seasonally free of ice and snow (Bockheim 2015). Many endemic species inhabit these ice-free terrestrial areas, where birds and marine mammals breed in the coastal zones (Chown and Convey 2007; Hughes 2010). Approximately 400 species of birds have been recorded from the Antarctic continent and oceanic waters north to approximately 40°S (Shirihai 2007). Vanstreels et al. (2020) recently summarized data on the biodiversity of ectoparasites associated with Antarctic and Subantarctic birds and reported 30 feather mite species from 28 bird species in this region.

King George Island is the largest of the South Shetland Islands at the northwest tip of the Antarctic Peninsula (Potapowicz et al. 2020). This island has six areas designated as the Antarctic Specially Protected Areas (ASPA), one of which is ASPA No. 171, located on the southeast coast of the Barton Peninsula. Approximately 5,000 pairs of two penguin species, *Pygoscelisantarcticus* (Forster) and *P.papua* (Forster), breed in ASPA No. 171, and 14 other bird species have been observed on the Barton Peninsula: *Chionisalbus* (Gmelin), *Larusdominicanus* Lichtenstein, *Stercorariusantarcticus* (Lesson), *S.maccormicki* Saunders, *Sternaparadisaea* Pontoppidan, and *St.vittata* Gmelin (Charadriiformes), *Leucocarbobransfieldensis* (Murphy) (Pelecaniformes), *Daptioncapense* (Linnaeus), *Fregettatropica* (Gould), *Fulmarusglacialoides* (Smith), *Macronectesgiganteus* (Gmelin), *Oceanitesoceanicus* (Kuhl) (Procellariiformes), *Eudypteschrysolophus* (Brandt), *Pygoscelisadeliae* (Hombron & Jacquinot) (Sphenisciformes) (Kim et al. 2005, 2014).

To date, no studies have been conducted on feather mites associated with birds living on the Barton Peninsula. In the present work, we report seven analgoid feather mites, including descriptions of two new species from the genera *Alloptes* and *Ingrassia*, found on four bird species on the Barton Peninsula of King George Island. Additionally, we provide DNA barcodes for the mitochondrial cytochrome c oxidase subunit I (COI) from these seven analgoid feather mite species.

## Materials and methods

### Material sampling

Mite samples were obtained from the Antarctic Shag (*L.bransfieldensis*), South Polar Skua (*S.maccormicki*), Wilson’s Storm Petrel (*O.oceanicus*), and three Snowy Sheatbills (*Ch.albus*) in the Barton Peninsula. The birds were captured using a hand net or loop according to ‘SKUAS Manual for Fieldworkers’ (PBEG 2003), and all birds were released after collecting the mites. Feather mites were collected using 3M ScotchMagicTape (3M, St. Paul, Minnesota, USA) from the wing, down, and tail feathers, and then immediately preserved in 70% ethanol for 3 h. The preserved samples were separated from Scotch tape under a dissecting microscope with a dissecting needle and then preserved in 95% ethanol. The collected mite specimens were cleared in 10% lactic acid for 24 h at room temperature and then mounted on microscope slides using PVA mounting medium (BioQuip, Rancho Dominguez, California, USA).

Descriptions of two new species are given according to standard formats used for the corresponding feather mite taxa (Mironov and Palma 2006; Mironov and Proctor 2008; Stefan et al. 2013; Hernandes et al. 2017). Terminology, idiosomal, and leg chaetotaxy follow Gaud and Atyeo (1996), with minor corrections for the coxal chaetotaxy by Norton (1998). All measurements are in micrometers (μm). All examined specimens are deposited at the National Institute of Biological Resources (NIBR), Korea. The classification and scientific names of birds follow Gill et al. (2021).

### DNA sequencing

Before preparing the microscopic slides, genomic DNA was extracted from one leg of each specimen using a Tissue DNA Purification Kit (Cosmogenetech Inc., Seoul, Korea) according to the manufacturer’s instructions. The COI barcode fragment was amplified using two universal primers: bcdF05 (5′-TTTTCTACHAAYCATAAAGATATTGC-3′) and bcdR04 (5′- TATAAACYTCDGGATGNCCAAAAAA-3′) under the following conditions: 2 min at 94 °C; 40 cycles at 98 °C for 15 s, 50 °C for 30 s, and 68 °C for 60 s; and a final extension at 68 °C for 5 min (Dabert et al. 2008). The amplified products were sequenced using an ABI3100 automated sequencer (Perkin Elmer, Foster City, California, USA). Sequence assembly, alignment, and trimming were performed using Geneious 8.1.9 software (Kearse et al. 2012). We obtained a 654 bp fragment sequence of the COI gene from two individuals per mite species.

## Systematic account

**Superfamily Analgoidea Trouessart & Mégnin, 1884**

### Family Alloptidae Gaud, 1957

#### Genus Alloptes

Taxon classificationAnimaliaSarcoptiformesAlloptidae

Canestrini, 1879

26AF2C63-CD40-508A-8806-1FBBECB9AA01

##### Notes.

*Alloptes* is one of the most specious genera of the family Alloptidae and currently includes about 50 described species (Gaud 1972; Vasyukova and Mironov 1991; Kivganov and Mironov 1992; Mironov and Palma 2006). All representatives of this genus are associated with birds of the order Charadriiformes, with exception of a questionable host association of *Alloptestubinarii* Dubinin, 1949 reported from several procellariiform hosts (Dubinin 1949). Gaud (1972) subdivided the genus into the three subgenera, *Alloptes* s. str., *Apodalloptes* Gaud, 1972, and *Conuralloptes* Gaud, 1972. Further, nearly a half of species of the subgenus Conuralloptes was arranged into a fourth subgenus, *Sternalloptes* Mironov, 1992 (in Kivganov and Mironov 1992). Three *Alloptes* species found on marine birds of the Barton Peninsular belong to three different dubgenera. Below we provide descimination features for these subgenera.

#### Subgenus Alloptes

Taxon classificationAnimaliaSarcoptiformesAlloptidae

Canestrini, 1879

708CDDDC-9991-58BB-9848-A8FCE0929D65

##### Notes.

The subgenus Alloptes s. str. currently includes three species and is characterized by the following features (Gaud 1952, 1972; Mironov 1996): in both sexes, seta *mG* of genu II is spiculiform; in males, the opisthosoma is roughly shaped as an equilateral triangle with terminal part strongly enlarged, setae *h3* are present, setae *ps2* are well developed (half as long as *f2*); in females, the opisthosoma is rounded, the opisthosomal lobes are not developed, idiosomal setae *ps1* and *f2* are present. Representatives of the subgenus are known from birds of the families Scolopacidae and Chionidae (Gaud 1952, 1957, 1972; Vasyukova and Mironov 1991). Five *Alloptes* species described by Dubinin (1952) from auks (Alcidae) could also belong to this subgenus, because these mites have filiform genual setae *mG*II and females have the opisthosoma rounded or with strongly abbreviated lobes, but all these species need re-investigation.

#### Alloptes (Alloptes) aschizurus

Taxon classificationAnimaliaSarcoptiformesAlloptidae

Gaud, 1952

6A000643-5A9E-5394-8E42-2300E27AB5E7

Alloptes aschizurus Gaud, 1952: 163–164, fig. 2; Atyeo and Peterson 1967: 98; 1970: 129.
Alloptes (Alloptes) aschizurus : Gaud 1972: 59.

##### Material examined.

3 males and 3 females (NIBR No. NIBRIV0000887146–NIBRIV0000887151) from *Chionisalbus* (Gmelin) (Charadriiformes, Chionidae), Antarctica, King George Island, Barton Peninsula, 62°14'16"S, 58°46'13"W, 8 January 2016, coll. Han Y.-D.

##### Remarks.

Alloptes (Alloptes) aschizurus was initially described from specimens collected from the Black-faced Sheathbill, *Chionisminor* (Hartlaub) on Kerguelen Island (Gaud 1952). Later, this mite species was found on the same host on Heard Island and on the Snowy Sheathbill, *Ch.albus*, on Greenwich Island and Gaston Islands (Atyeo and Peterson 1967). As for all members of the subgenus Alloptes s. str., this mite species is characterized by the following features: in both sexes, genual setae *mG*II are spiculiform; in males, the opisthosoma is shaped as an equilateral triangle with a strongly enlarged posterior end; in females, the posterior end of the opisthosoma is rounded, and the opisthosomal lobes are not developed (Gaud 1972; Vasyukova and Mironov 1991; Mironov and Hernandes 2020). Alloptes (A.) aschizurus is distinguished from the closest species of the subgenus, A. (A.) tringae (Grube, 1859) [widely known under the junior synonym A. (A.) crassipes (Canestrini, 1878)] in having the following features. In both sexes, the length of the idiosoma is approximately 500 long (vs approximately 450 in *A.tringae*), and trochanteral setae *sR*III are equal to or slightly longer than the trochanters III (vs distinctly shorter than the trochanters) (Gaud 1952; Atyeo and Peterson 1967; Vasyukova and Mironov 1991; Mironov and Hernandes 2020).

##### Molecular data.

The COI sequences were obtained from two individuals and deposited in GenBank with accession numbers MZ489637 and MZ489638.

#### Subgenus Conuralloptes

Taxon classificationAnimaliaSarcoptiformesAlloptidae

Gaud, 1972

EDEC4D17-C03A-5CF9-AB17-3B33FCCD201D

##### Notes.

The subgenus Conurlloptes currently includes 23 species and is characterized by the following features (Gaud 1972; Vasyukova and Mironov 1991): in both sexes, seta *mG* of genu II is short spine-like with widely rounded apex; in males, the opisthosoma is triangular, gradually narrowed posteriorly and without posterior enlargement, idiosomal setae *h3* are absent, setae *ps2* are strongly reduced (barely distinct); in females, opisthosoma with well-developed opisthosomal lobes, setae *ps1* and *f2* are present. This subgenus is known from birds of the families Chionidae, Pedionomidae, Recurvirostridae, and Scolopacidae in the order Charadriiformes (Gaud 1972; Vasyukova and Mironov 1991; Mironov and Palma 2006).

#### Alloptes (Conuralloptes) chionis

Taxon classificationAnimaliaSarcoptiformesAlloptidae

Atyeo & Peterson, 1967

1BA07416-CF2C-5457-8BB0-25CA010698AF

Alloptes chionis Atyeo & Person, 1967: 98, figs 1–4; 1970: 129–130, figs 15–17.
Alloptes (Conuralloptes) chionis : Mironov 2007: 619.

##### Material examined.

3 males and 3 females (NIBR No. NIBRIV0000887152–NIBRIV0000887157) from *Chionisalbus* (Gmelin) (Charadriiformes, Chionidae), Antarctica, King George Island, Barton Peninsula, 62°14'3"S, 58°46'56"W, 13 January 2016, coll. Han Y.-D.

##### Remarks.

Alloptes (Conuralloptes) chionis was described from specimens collected from *Ch.minor* (type host) on Heard Island and was also found on *Ch.albus* from the Gaston Islands (Atyeo and Person 1967). When this mite was described, the genus *Alloptes* had not yet been subdivided into subgenera. Gaud (1972) established three subgenera in this genus but did not consider the taxonomic position of this species. Mironov (2007) placed this mite in the subgenus Conuralloptes based on the following characters: in both sexes, genual setae *mG*II are shaped as short and thick spines with bluntly rounded apices; in males, the opisthosoma is not enlarged apically, and idiosomal setae *h3* are absent; in females, the idiosomal setae *ps1* and *f2* are present. The males of A. (C.) chionis can be distinguished from other species of the subgenus Conuralloptes by the following combination of features: the anterior margin of the hysteronotal shield is slightly convex, the pregenital sclerites are free from each other and almost parallel, the adanal shields are C-shaped, and macrosetae *h2* are flattened and slightly widened in the medial part (Atyeo and Peterson 1967, 1970).

##### Molecular data.

The COI sequences were obtained from two individuals and deposited in GenBank with accession numbers MZ489639 and MZ489640.

#### Subgenus Sternalloptes

Taxon classificationAnimaliaSarcoptiformesAlloptidae

Mironov, 1992

2F366733-BBA4-518E-98EE-B5AF018E2321

##### Notes.

The subgenus Sternalloptes includes about 20 species and is characterized by the following features (Kivganov and Mironov 1992; Mironov 1996): in both sexes, seta *mG* of genu II is shortspine-like with widely rounded apex; in males, the opisthosoma is triangular, gradually narrowed posteriorly and with noticeable terminal enlargement, idiosomal setae *h3* are present or absent, setae *ps2* are strongly reduced; in females, the opisthosoma with well-developed opisthosomal lobes, idiosomal setae *ps1* and *f2* are absent. Common hosts of the subgenus Sternalloptes are birds of the families Laridae and Stercorariidae in the order Charadriiformes (Gaud 1976; Vasyukova and Mironov 1991; Kivganov and Mironov 1992; Mironov and Kivganov 1993).

#### Alloptes (Sternalloptes) antarcticus sp. nov.

Taxon classificationAnimaliaSarcoptiformesAlloptidae

FEEB3F6A-14DB-5FB8-9DDB-8A2AF1C296BB

http://zoobank.org/5C7680DD-1773-4EE8-B7C5-D99285367323

##### Type material.

Male holotype (NIBR No. NIBRIV0000887158), 3 males and 4 females paratypes (NIBR No. NIBRIV0000887159–NIBRIV0000887164) from *Stercorariusmaccormicki* Saunders (Charadriiformes, Stercorariidae), Antarctica, King George Island, Barton Peninsula, 62°14'2"S, 58°46'20"W, 2 January 2016, coll. Han Y.-D.

##### Description.

**Figure 1.**
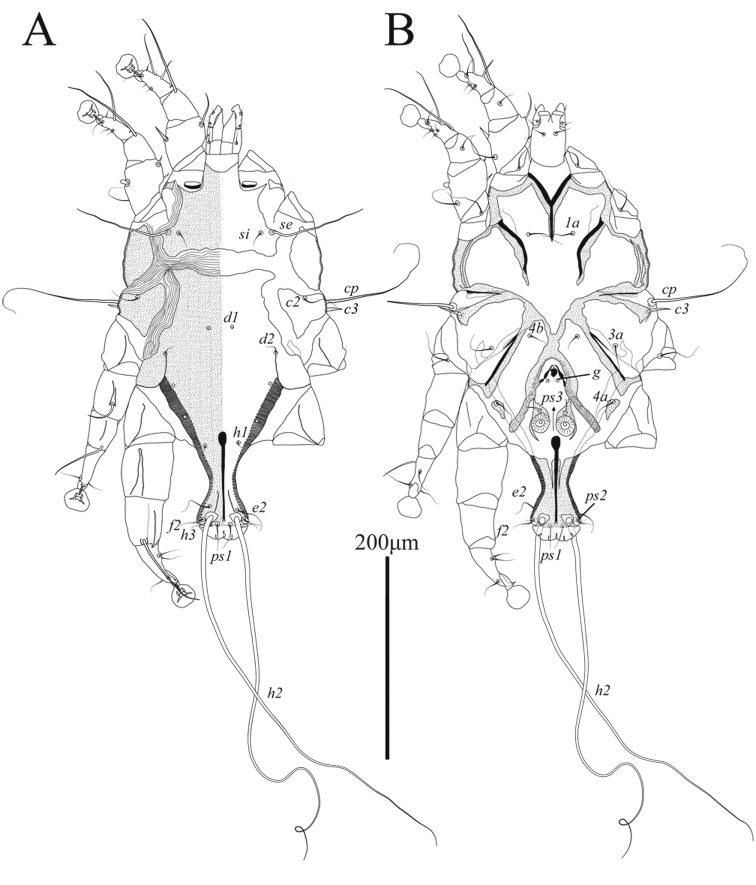
Alloptes (Sternalloptes) antarcticus sp. nov., male **A** dorsal view **B** ventral view.

**Male** (Figs 1, 3A–E, 4A, B; holotype, range for 3 paratypes in parentheses): idiosoma, length × width, 370 (340–365) × 200 (175–205). Length of hysterosoma 228 (213–243). Prodorsal shield (Figs 1A, 4A): length 80 (78–80), width at posterior margin 114 (102–118), posterolateral corners truncate, posterior margin slightly concave. External scapular setae *se* situated on posterolateral extensions of prodorsal shield near their anterior margins. Hysteronotal shield: greatest length 255 (235–258), width at anterior margin 100 (98–108), anterior margin slightly concave, lateral margins without incisions at bases of setae *d2* and fused ventrally with bases of epimerites IV. Length between prodorsal and hysteronotal shields along midline 31 (18–22). Dorsal setae *c2* 32 (33–36) long, shorter than trochanters III (Fig. 4C). Subhumeral setae *c3* narrowly lanceolate, 23 (19–23) × 2 (3). Posterior part of opisthosoma gradually expanded at posterior end, width at level of setae *h2* 50 (46–53). Length of interlobar septum 82 (80–86). Terminal lamella with three pairs of festoons; incision between inner pair narrow slit-like or inner festoons slightly overlapping. Setae *h3* present, setae *ps2* distinct. Setae *h2* cylindrical, not expanded in medial part. Dorsal measurements: *se*:*se* 118 (96–106), *c2*:*d2* 55 (50–57), *d2*:*ps1* 165 (158–168). Bases of trochanters I, II flanked by narrow sclerotized bands connecting bases of corresponding epimerites (Fig. 1B). Pregenital sclerites fused as a Y, their anterior ends connected to inner ends of epimerites IIIa, posterior end fused with paragenital arch. Coxal fields III and IV closed. Length of genital-anal field 163 (158–168). Genital arch: 17 (15–17) × 20 (19–20). Coxal setae *4b* situated anterior to level of setae *3a*. Setae *4a* surrounded by sclerites of irregular form. Ventral measurements: *3a*:*4b* 10 (9–13), *4b*:*g* 45 (43–43), *4b*:*4a* 63 (60–65), *g*:*ps3* 30 (31–35), *ps3*:*ps1* 110 (109–117), *4a*:*4a* 120 (110–112). Setae *mG* of genua I thin spine-like with acute apex, setae *mG*II shaped as thick spine with bluntly rounded apex. Legs IV 203 (193–203) long. Distal margin of tibia IV with small spine. Tarsus IV 54 (49–55) long, with claw-like apex; setae *d* and *e* small spine-like, seta *e* situated near tarsal apex, seta *d* at level of seta *f*, setae *r* and *w* in basal one-third of the segment (Fig. 3A–C).

**Figure 2.**
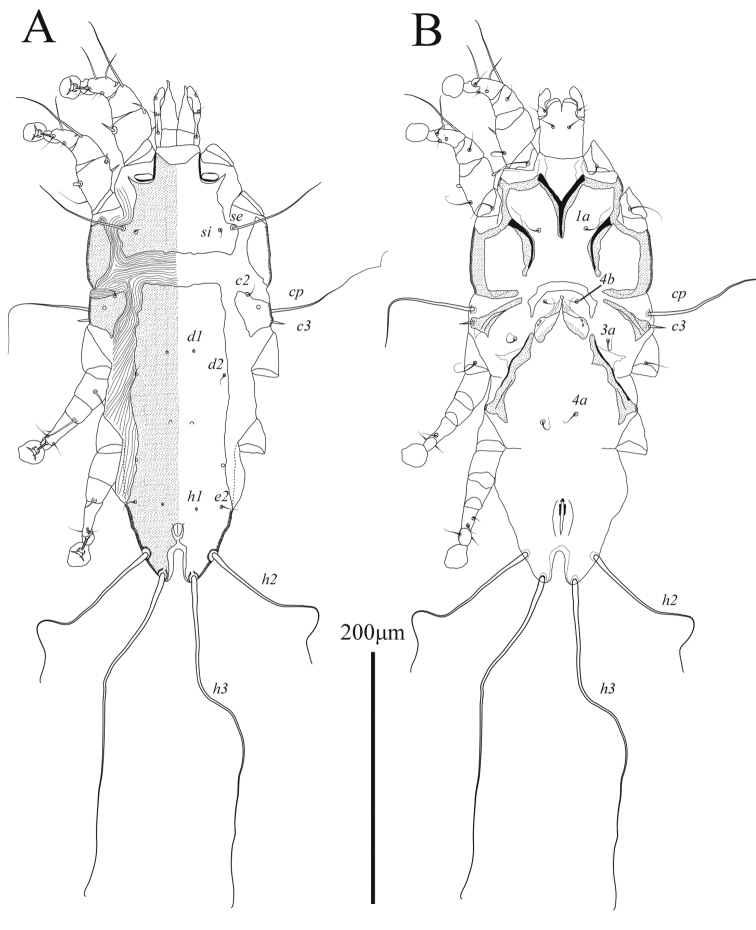
Alloptes (Sternalloptes) antarcticus sp. nov., female **A** dorsal view **B** ventral view.

**Figure 3.**
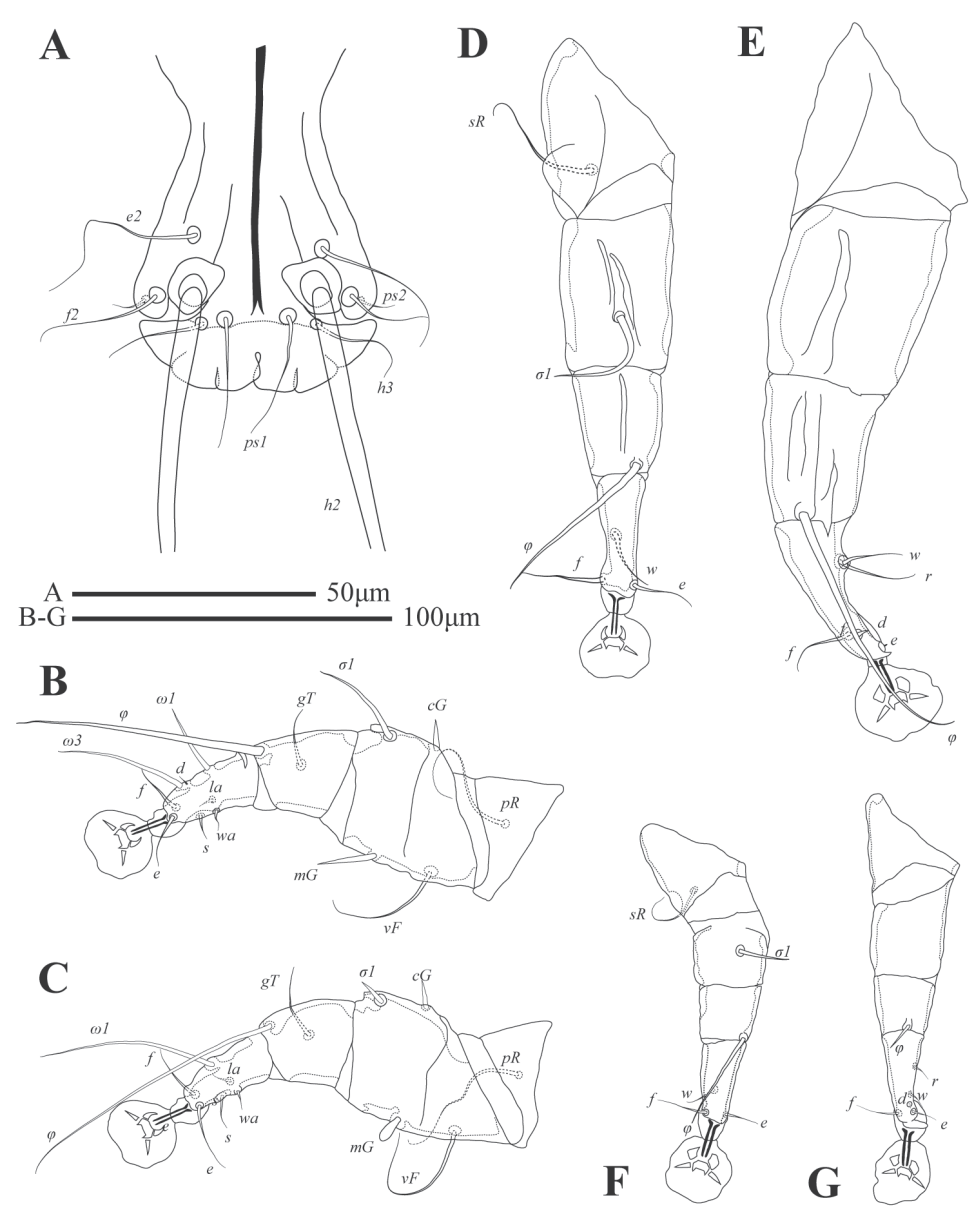
Alloptes (Sternalloptes) antarcticus sp. nov., details **A** opisthosoma of male, dorsal view **B** leg I of male **C** leg II of male **D** leg III of male **E** leg IV of male **F** leg III of female **G** leg IV of female.

**Figure 4.**
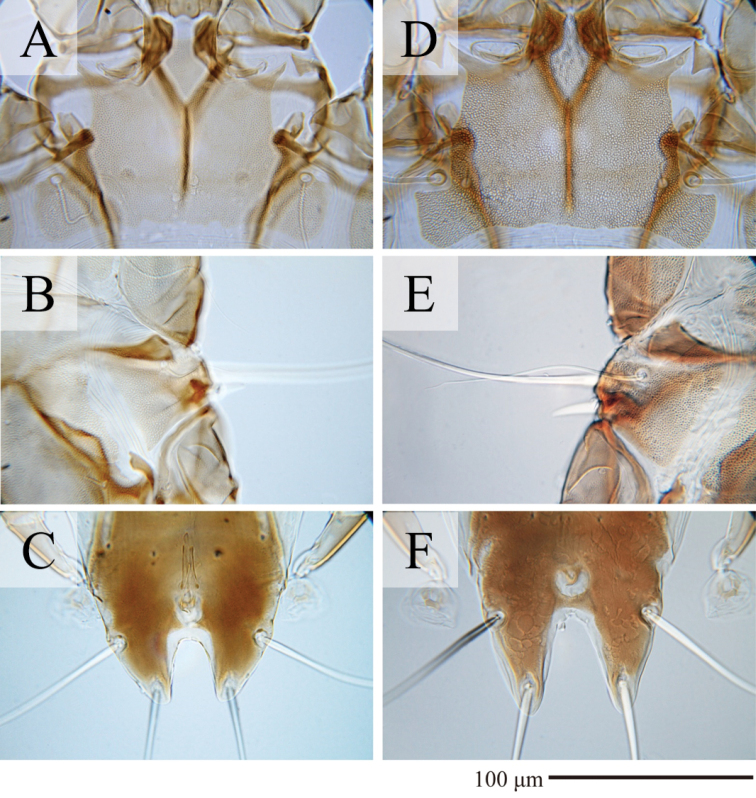
*Alloptes* species **A–C**Alloptes (Sternalloptes) antarcticus sp. nov. **D–F**A. (S.) catharacti**A, D** prodorsal shield of males **B, E** humeral shield of males **C, F** opisthosomal lobes of females.

**Female** (Figs 2, 4F, G, 4C; range for 4 paratypes): idiosoma, length × width, 350–360 × 148–153 (Fig. 2A). Hysterosoma 238–250 long. Prodorsal shield: shaped as in male, 80–83 × 85–90. Setae *c2* 10–14 long, shorter than trochanters III. Setae *c3* lanceolate, 13–15 × 2–3. Hysteronotal shield: 233–238 × 60–62, anterior margin straight or slightly concave, surface without ornamentation. Setae *h1* and *e2* situated at same transverse level. Setae *f2* and *ps1* absent. Distance between prodorsal and hysteronotal shields along midline 23–33. Supranal concavity ovate, opened posteriorly, delimited from terminal cleft by short extensions. Opisthosomal lobes well developed, approximately as long as wide at base, terminal cleft as an inverted U, 24–30 long, 12–20 wide (Fig. 4C). Anterior end of supranal concavity extending slightly beyond level of setae *h2*. Dorsal measurements: *se*:*se* 83–89, *c2*:*d2* 64–67, *d2*:*e2* 104–106, *e2*:*h2* 39–43, *h2*:*h3* 19–20, *h2*:*h2* 55–60, *h3*:*h3* 25–31. Bases of trochanters I, II flanked by narrow sclerotized bands connecting bases of corresponding epimerites (Fig. 2B). Epimerites IVa barely distinct. Epigynum bow-shaped, 24–27 × 55–59. Legs IV with ambulacral discs reaching level of insertions of setae *h2* (Figs 2, 3F, G).

##### Differential diagnosis.

Among 18 previously known species in the subgenus Sternalloptes (Kivganov and Mironov 1992; Mironov and Kivganov 1993; Kivganov 1996; Hernandes et al. 2017), the new species Alloptes (S.) antarcticus sp. nov. is most similar to A. (S.) catharacti Mironov, 1991 found on the same host, *S.maccormicki* from Mirny station (Queen Mary Land, Antarctica), in having setae *c3* lanceolate and short (shorter that trochanters III), the pregenital sclerite fused into a Y connecting the tips of epimerites IIIa and the apex of the paragenital arch, and setae *h2* not expanded (Mironov 1991). Alloptes (S.) antarcticus sp. nov. differs from A. (S.) catharacti in having the following characteristics: in both sexes, the external scapular setae *se* are situated on the posterolateral extensions of the prodorsal shield; in males, the dorsal setae *c2* (32–36) are approximately 1.5 times longer than setae *c3* (19–23) and shorter than trochanters III (Fig. 4A, B); in females, the terminal cleft is shorter (24–30 long), and the supranal concavity is open posteriorly into the terminal cleft (Fig. 4C). In both sexes of A. (S.) catharacti, setae *se* are situated on the soft tegument near the anterior margin of the posterolateral extensions of the prodorsal shield; in males, the dorsal setae *c2* (63–93) are 2–3 times longer than setae *c3* (24–29) and exceed the length of trochanters III (Fig. 4D, E); in females, the terminal cleft is longer (38–48), and the supranal concavity is separated from the terminal cleft (Mironov 1991) (Fig. 4F).

##### Remark.

The comparative material of A. (S.) catharacti used here to illustrate morphological differences was collected from the same host species, *S.maccormicki*, at Jangbogo station, Terra Nova Bay, Antarctica, in 2016, by Ji-Yong Lee.

##### Molecular data.

The COI sequences were obtained from two individuals and deposited in GenBank with accession numbers MZ489641 and MZ489642.

##### Etymology.

The specific name refers to the geographical range of the type host.

### Family Avenzoariidae Oudemans, 1905

**Subfamily Bonnetellinae Atyeo & Gaud, 1981**

#### Genus *Scutomegninia* Dubinin, 1951

**Subgenus Scutomegninia Dubinin, 1951**

##### Scutomegninia (Scutomegninia) subantarctica

Taxon classificationAnimaliaSarcoptiformesAvenzoariidae

Mironov, 2000

B7B05B9D-A6EC-5388-AFA7-C5D3984B77DA

Scutomegninia phalacrocoracis : Atyeo & Peterson 1967: 100, figs 5–8; 1970: 150, figs 68–70.
Scutomegninia subantarctica Mironov, 1990: 53, nom. nudum.
Scutomegninia (Scutomegninia) subantarctica : Mironov 2000: 14–18, fig. 5.

###### Material examined.

1 male and 3 females (NIBR No. NIBRIV0000887165–NIBRIV0000887168) from *Leucocarbobransfieldensis* (Murphy) (Suliformes, Phalacrocoracidae), Antarctica, King George Island, Barton Peninsula, 62°14'4"S, 58°46'52"W), 8 January 2016, coll. Han Y.-D.

###### Remarks.

Mites of the genus *Scutomegninia*, collected from the Imperial Shag, *Leucocarboatriceps* (King) (= *Phalacrocoraxatriceps*) in Maipo Island (Buls Bay on Brabant Island, Palmer Archipelago, Antarctica) by Atyeo and Peterson (1967), were originally identified as *S.phalacrocoracis* (Dubinin and Dubinina, 1940). Furthermore, Atyeo and Peterson (1970) reported S. (S.) phalacrocoracis from *Leucocarbogeorgianus* (Lönnberg) (= *P.atricepsgeorgianus*) from Bird Island, South Georgia. Later, Mironov (1990, 2000) described specimens from the Palmer Archipelago as a separate species, S. (S.) subantarctica. According to the present taxonomic view, *Phalacrocoraxatriceps* belongs to the genus *Leucocarbo* and is split into several separate species restricted to particular areas of the Antarctic and subantarctic regions (Gill et al. 2021). Taking into consideration this concept, S. (S.) subantarctica reported by previous researchers (Atyeo and Peterson 1967, 1970) were collected from the Antarctic Shag, *L.bransfieldensis* (Antarctic Peninsula and Palmer Archipelago), and the South Georgia Shag, *L.georgianus* (South Georgia).

Scutomegninia (S.) subantarctica belongs to the *phalacrocoracis* group (species associated with Phalacrocoracidae and Anhingidae), and it is most similar to S. (S.) pygmaea Mironov, 1990. It differs from S. (S.) pygmaea and other species of the *phalacrocoracis* group by the following combination of characters in males: the terminal ends of the interlobar membrane have a small spine-like process; the lateral adanal shields have acute posterior ends, while the medial adanal shields have the posterior ends bluntly rounded; the anteromedial ends of adanal apodemes are rounded; setae *s* of tarsus III are spine-like, strongly attenuate apically, and bear two small denticles; the terminal cleft is 1.8–2 times longer than wide; and the incision in the interlobar membrane extends to the level of setae *h2* (Mironov 1990, 2000).

###### Molecular data.

The COI sequences were obtained from two individuals and deposited in GenBank with accession numbers MZ489643 and MZ489644.

#### Genus *Zachvatkinia* Dubinin, 1949

##### Zachvatkinia hydrobatidii

Taxon classificationAnimaliaSarcoptiformesAvenzoariidae

Dubinin, 1949

58024331-6923-5782-A491-6448F8D0C6AA

Zachvatkinia hydrobatidii Dubinin, 1949: 219, figs 9b, 10b; 1952, 256; Atyeo and Peterson 1967: 101, figs 9–12; 1970: 146, figs 61–63; Mironov 1989: 110–115, figs 5, 7, 8.

###### Material examined.

3 males and 3 females (NIBR No. NIBRIV0000887169–NIBRIV0000887174) from *Oceanitesoceanicus* (Kuhl) (Procellariiformes, Oceanitidae), Antarctica, King George Island, Barton Peninsula, 62°14'15'S, 58°46'28"W, 9 January 2016, coll. Han Y.-D.

###### Remarks.

*Zachvatkiniahydrobatidii* was described by Dubinin (1949) based on specimens collected from the Wilson’s Storm Petrel, *O.oceanicus* in Massachusetts (USA), and also from 10 other storm petrels of the genera *Fregetta*, *Garrodia*, *Pelagodroma* (Oceanitidae), and *Oceanodroma* (Hydrobatidae) from various parts of the world. Mironov (1989) re-examined most of this material and referred to this mite species only the specimens from the oceanitids *O.oceanicus* and *F.tropica*. In Antarctica, *Z.hydrobatidii* was previously reported from *O.oceanicus*, *F.tropica*, and *Pagodromanivea* (Forster) (Procellariidae) (Atyeo and Peterson 1967, 1970). The record from the procellariid host seems to be questionable. *Zachvatkiniahydrobatidii* is very close to *Z.oceanodromae* Mironov, 1989 associated with storm petrels of the genus *Oceanodroma*, and differs in having the following features: in males, the genital arch is shaped as a completely closed ring, and the distance between setae *ps1* and *h3* is less than 40; in females, the posterior margin of the opisthosoma between the terminal extensions is not sclerotized, and setae *e1* are situated on the inner margins of the lateral hysteronotal shields (Mironov 1989).

###### Molecular data.

The COI sequences were obtained from two individuals and deposited in GenBank with accession numbers MZ489645 and MZ489646.

##### Zachvatkinia stercorarii

Taxon classificationAnimaliaSarcoptiformesAvenzoariidae

Dubinin, 1952

E0AD9F61-60F9-5B0B-B80F-8A16F17B9B1B

Zachvatkinia stercorarii Dubinin, 1949: 227, fig. 12, nom. nudum, 1952: 255, figs 1, 2; Atyeo and Peterson 1967: 103, 1970: 147; Mironov 1989: 100–111, figs 3, 7, 8.

###### Material examined.

3 males and 3 females (NIBR No. NIBRIV0000887175–NIBRIV0000887180) from *Stercorariusmaccormicki* Saunders (Charadriiformes, Stercorariidae), Antarctica, King George Island, King Sejong station, Barton Peninsula, 62°14'2"S, 58°46'20"W, 21 January 2016, coll. Han Y.-D.

###### Remarks.

*Zachvatkiniastercorarii* was described by Dubinin (1952) based on specimens collected from three species of skuas or jaegers, *Stercorariuspomarinus* (Temminck) (type host), *S.parasiticus* (Linnaeus), and *S.longicaudus* Vieillot, from Wrangel Island, Russia. Furthermore, it was shown that mites from *S.parasiticus* and *S.longicaudus* belong to a separate species, *Z.isolata* Mironov, 1989 (Mironov 1989; Dabert et al. 2015). In Antarctica, *Z.stercorarii* was previously reported from *S.antarcticus* (Lesson) from Adelaide Island and the Palmer Archipelago and from *S.maccormicki* from Cape Hallett, Haswell Islands, Ross Island, and Victoria Land (Atyeo and Peterson 1967, 1970).

Although *Z.stercorarii* and *Z.isolata* are associated with birds in the order Charadriiformes, these mite species belong to the *puffini* species group, which is characterized by a single dorsobasal spine on tarsus IV in males and setae *d1* situated off the lateral hysteronotal shields in females (Mironov 1989). All remaining species of the *puffini* group are associated with Procellariiformes, while other representatives of the genus *Zachvatkinia* associated with Charadriiformes belong to the *sternae* species group. It was hypothesized that the common ancestors of *Z.stercorarii* and *Z.isolata* were probably transferred from some procellariiform hosts to the ancestor of the family Stercorariidae (Dabert and Mironov 1999).

*Zachvatkiniastercorarii* can be clearly distinguished from *Z.isolata* in having the following features: in males, the bases of genital setae *g* are adjacent (vs distant from each other); in females, the posterior margin of the prodorsal shield is just slightly convex (vs strongly convex), and the lateral margins of this shield have small incisions posterior to the bases of setae *se* (vs smooth and without incisions) (Mironov 1989).

###### Molecular data.

The COI sequences were obtained from two individuals and deposited in GenBank with accession numbers MZ489647 and MZ489648.

### Family Xolalgidae Dubinin, 1953

**Subfamily Ingrassiinae Gaud & Atyeo, 1981**

#### Genus Ingrassia

Taxon classificationAnimaliaSarcoptiformesXolalgidae

Oudemans, 1905

B61E0787-E5E0-5D89-9B08-33B618416369

##### Notes.

The genus *Ingrassia* is the most specious genus within the subfamily Ingrassiinae, including 28 species up to now (Gaud 1972; Vasyukova and Mironov 1991; Mironov and Proctor 2008; Stefan et al. 2013). Representatives of the genus have been recorded on hosts from six orders of aquatic birds: Anseriformes, Charadriiformes, Pelecaniformes, Podicipediformes, Procellariiformes, and Sphenisciformes. Identification keys to species of *Ingrassia* are available only for those associated with birds in the order Charadriiformes in Africa (Gaud 1972) and northern Eurasia (Vasyukova and Mironov 1991). To date, only six species of the genus *Ingrassia* have been recorded from procellariiform birds (Stefan et al. 2013).

#### Ingrassia chionis sp. nov.

Taxon classificationAnimaliaSarcoptiformesXolalgidae

72B50C50-01BF-58DB-AE3B-C3FE4674047F

http://zoobank.org/D6242043-BF80-4B66-B6AB-41CB5B073134

##### Type material.

Male holotype (NIBR No. NIBRIV0000887181), 2 males and 3 females paratypes (NIBR No. NIBRIV0000887182–NIBRIV0000887186) from *Chionisalbus* (Gmelin) (Charadriiformes, Chionidae), Antarctica, King George Island, Barton Peninsula, 62°14'13"S, 58°46'33"W, 11 January 2016, coll. by Han Y.-D.

##### Description.

**Figure 5.**
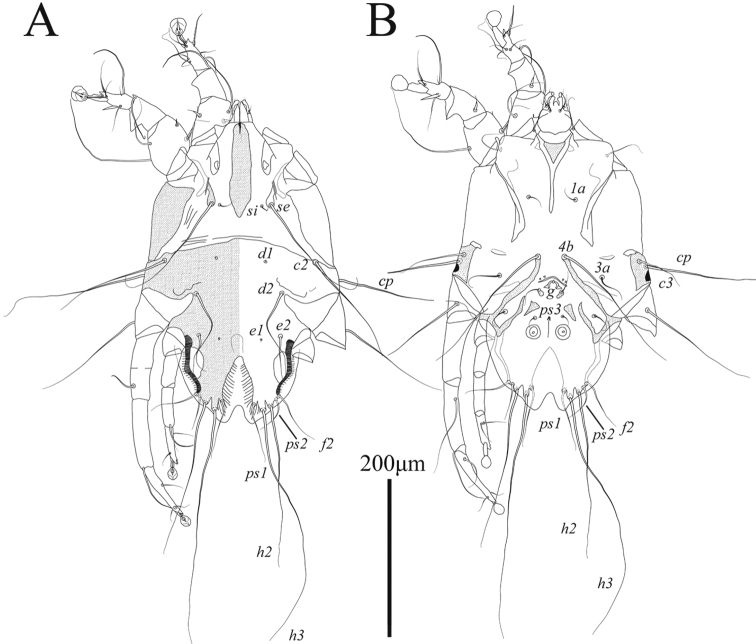
*Ingrassiachionis* sp. nov., male **A** dorsal view **B** ventral view.

**Male** (Figs 5, 7A–D; holotype, range for 2 paratypes in parentheses): length of idiosoma from anterior end to bases of setae *h3* 350 (350–355), greatest width 220 (230–240), length of hysterosoma 175 (173–175). Prodorsal shield: narrow longitudinal plate with almost parallel lateral margins and acute posterior end extending beyond level of scapular setae *se*; length along midline 113 (118), greatest width 28 (27–29); anterior end with short longitudinal ridge about 1/8^th^ the length of shield (Fig. 5A). Setae *se* and *si* at same transverse level, bases of setae *se* situated on teardrop-shaped sclerites and separated by 69 (73–77). Scapular shields wide, inner margins slightly convex, without suprategumental extensions. Hysteronotal shield: anterior margin convex, length of shield from anterior end to bases of setae *h3* 213 (205–210). Setae *c2* and *d2* represented by macrosetae, 150 (150–150) and 110 (95–110) long, respectively; both pairs approximately 1.5 time shorter than humeral macrosetae *cp.* Opisthosomal lobes slightly longer than wide at base. Supranal concavity ovate, poorly outlined, separated from terminal cleft. Terminal cleft semi-ovate in shape, slightly narrowed anteriorly; length of terminal cleft from anterior end to bases of setae *h3* 63 (58–62), greatest width 40 (41–42). Terminal membranous extensions on lobar apices short and widely rounded, length from bases of setae *h3* to apices of terminal extensions 20 (18–19), width of extensions at base 32 (27–28), length of incision between extensions 22 (19–20). Setae *ps1* situated approximately at level of setae *h2*. Distance between dorsal setae: *c2*:*c2* 183 (180–188), *c2*:*d2* 33 (37–38), *d2*:*e2* 52 (50–53), *e2*:*h3* 88 (83–84), *h3*:*h3* 59 (55–57), *ps1*:*ps1* 39 (36–37).

Sternum about half as long as total length of epimerites I (Fig. 5B). Anterior ends of epimerites IIIa free, widely separated from each other. Setae *4b* situated on anterior ends of epimerites IIIa and almost extending to mid-length of opisthosomal lobes. Pregenital apodeme (epiandrum) small bow-shaped, 10 (7–9) long, 35 (32–38) wide. Genital apparatus 13 (10–12) long and 27 (27–28) wide. Setae *g* situated on small genital shields. Adanal shields triangular, situated anterolateral to setae *ps3*. Epimerites IVa long, almost completely enclosing coxal fields IV. Central part of coxal fields IV not sclerotized. Diameter of adanal suckers 19 (19–20). Distance between ventral setae: *4b*:*4b* 33 (35–36), *4b*:*3a* 24 (27–29), *4b*:*g* 41 (42–43), *g*:*ps3* 29 (32–33), *ps3*:*h3* 97 (93–95).

Tarsi I, II each with short apicodorsal extension. Tibiae I, II with well-developed ventral spine-like processes (Fig. 7A, B). Seta *s* of tibia II spiculiform. Femorogenu II with thick spine-like retrograde apophysis. Tibia III with small angular apical extension bearing base of solenidion *φ*. Length of tarsus III 81 (78–79). Tarsus IV with finger-like apical extension; modified setae *d*, *e* short spiculiform, seta *e* situated on tarsal apex, seta *d* subapical (Fig. 7C). Legs IV excluding pretarsus 55 (51–58) long, with tarsus and distal half of genu extending beyond level of lobar apices (bases of setae *h3*) (Figs 5, 7D).

**Figure 6.**
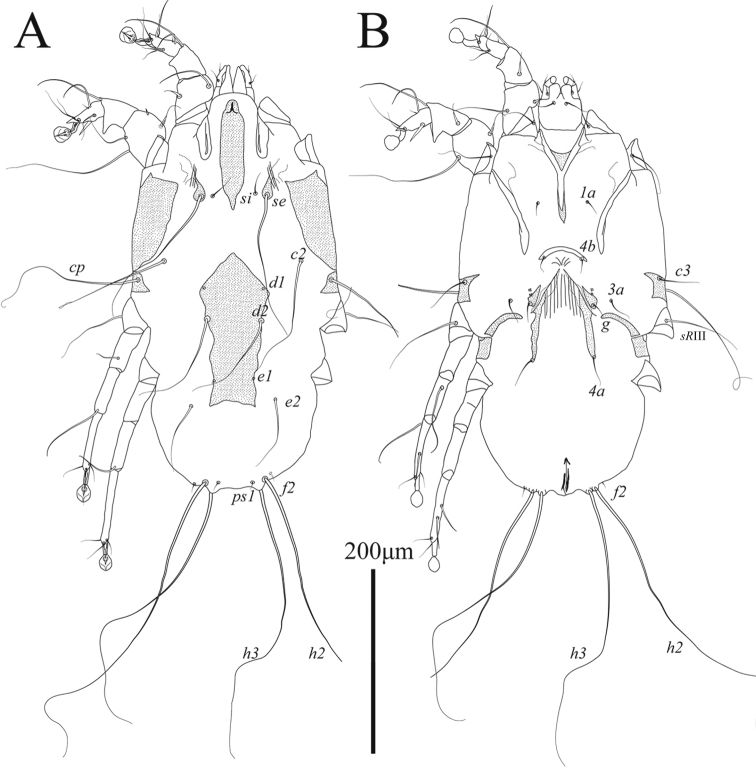
*Ingrassiachionis* sp. nov., female **A** dorsal view **B** ventral view.

**Figure 7.**
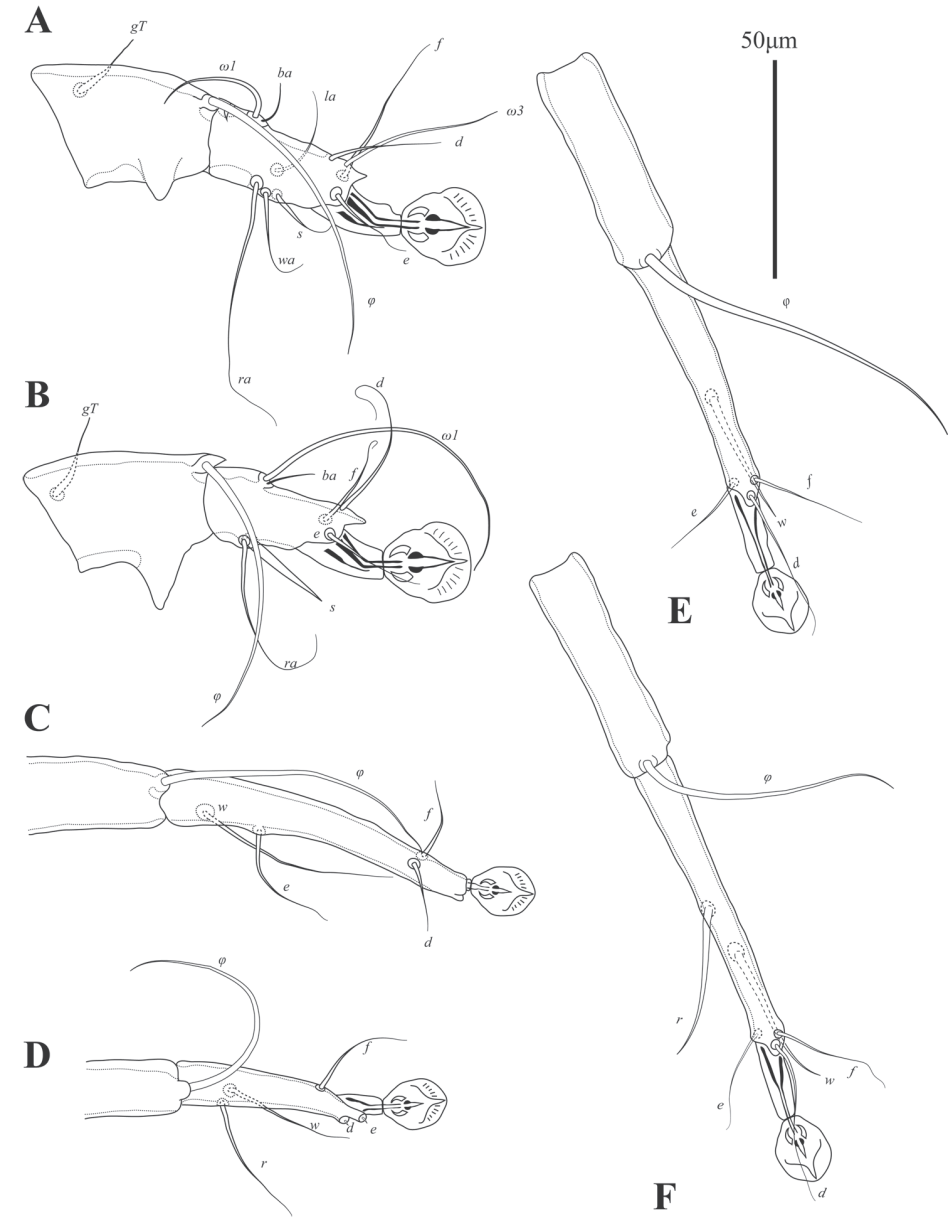
*Ingrassiachionis* sp. nov., legs **A** tibia and tarsus I of male **B** tibia and tarsus II of male **C** tibia and tarsus III of male **D** tibia and tarsus IV of male **E** tibia and tarsus III of female **F** tibia and tarsus IV of female.

**Female** (Figs 6, 7E, F; range for 3 paratypes): length of idiosoma 400–435, greatest width 225–250, length of hysterosoma 220–238. Prodorsal shield: shaped approximately as in male, length 118–123, greatest width 30–33, anterior end with short longitudinal ridge about 1/8^th^ the length of shield (Fig. 6A). Setae *se* and *si* at same transverse level; bases of setae *se* situated on teardrop-shaped sclerites and separated by 75–82. Scapular shields wide, with smooth inner margin. Humeral shields well developed, without anteromesal extensions. Setae *c3* short, slightly longer than trochanters III. Hysteronotal shield: large longitudinal plate occupying median part of hysterosoma; anterior part slightly widened; anterior margin right-angular, extending to or beyond level of setae *c2*; lateral margins unevenly sinuous; posterior margin truncate or slightly concave, extending to level of setae *e2*; greatest length 158–168, greatest width 85–93. Setae *c2*, *d2*, and *e2* represented by macrosetae, 139–151, 112–131 and 81–93 long, respectively. Setae *d1*, *d2*, and *e1* situated on hysteronotal shield, setae *c2*, *e2* situated on striated tegument. Distance between dorsal setae: *c2*:*d2* 63–73, *d2*:*e2* 84–87, *e2*:*h3* 82 87, *h3*:*h3* 52–70.

Sternum about half as long as epimerites I. Epigynum thick and is bow-shaped, 13–22 long, 58–64 wide, with tips bearing bases of setae *4b*. Apodemes of oviporus long, their posterior ends long and narrow, encompassing bases of setae *4a* (Fig. 6B). Setae *4b*, *g*, *3a*, and *4a* short, not exceeding length of femorogenua III, IV. Setae *h3* approximately two-thirds the length of setae *h2*.

Legs I, II as in the male. Legs IV with tarsus extending beyond posterior end of opisthosoma. Tarsi III, IV without apical spines, length of tarsi III, IV 60–61 and 72–74, respectively. Setae *sR*III subequal to combined length of corresponding femur, genu, and tibia. Seta *w* of tarsus III and setae *r*, *w* of tarsus IV spiculiform (Figs 6, 7).

##### Differential diagnosis.

The new species *Ingrassiachionis* sp. nov. belongs to a group of species associated with the Charadriiformes and is characterized by a retrograde spine-like apophysis on femorogenu II in both sexes (Gaud 1972; Vasyukova and Mironov 1991). Among this species grouping, the new species is most similar to *I.tringae* Vitzthum, 1922 (= *I.minuta* Gaud, 1972) described from *Calidrisminuta* (Leisler) (Scolopacidae) in having the following features: in males of both species, the opisthosomal lobes are relatively short, equal to or slightly longer than wide at the bases, and the supranal concavity is completely separated from the terminal cleft; in females, the hysteronotal shield is shaped as a large longitudinal plate occupying the median area of the hysterosoma. *Ingrassiachionis* is distinguished from *I.tringae* by the following features: in both sexes, the prodorsal shield is narrow, parallel-sided, with the width about one-third the distance between setae *se*, and the posterior end of this shield is tapering; in males, the terminal cleft is semi-ovate, narrowed only in the anterior end, and tibia III bears a small apical spine of rectangular shape; in females, the anterior margin of the hysteronotal shield is right-angled and extends to the level of setae *c2*, and the posterior margin of this shield is truncate and extends to the level of setae *e2*. In both sexes of *I.tringae*, the prodorsal shield is a longitudinal plate widened posteriorly, with its greatest width equal to or larger than the halfway between setae *se*, and the posterior margin is widely rounded; in males, the anterior half of the terminal cleft is strongly narrowed, and tibia III bears a pointed apical spine; in females, the anterior margin of the hysteronotal shield is semi-ovate and does not extend to the level of setae *c2*, and the posterior margin of this shield is concave and extends beyond the level of setae *e2*.

##### Molecular data.

The COI sequences were obtained from two individuals and deposited in GenBank with accession numbers MZ489649 and MZ489650.

##### Etymology.

The specific name is taken from the generic name of the type host and is a noun in apposition.

## Supplementary Material

XML Treatment for Alloptes

XML Treatment for Alloptes

XML Treatment for Alloptes (Alloptes) aschizurus

XML Treatment for Conuralloptes

XML Treatment for Alloptes (Conuralloptes) chionis

XML Treatment for Sternalloptes

XML Treatment for Alloptes (Sternalloptes) antarcticus

XML Treatment for Scutomegninia (Scutomegninia) subantarctica

XML Treatment for Zachvatkinia hydrobatidii

XML Treatment for Zachvatkinia stercorarii

XML Treatment for Ingrassia

XML Treatment for Ingrassia chionis
